# MicroRNA-200a activates Nrf2 signaling to protect osteoblasts from dexamethasone

**DOI:** 10.18632/oncotarget.20452

**Published:** 2017-08-24

**Authors:** Sai Zhao, Li Mao, Shou-Guo Wang, Feng-Li Chen, Feng Ji, Hao-Dong Fei

**Affiliations:** ^1^ Department of Pediatrics, Huai'an First People's Hospital, Nanjing Medical University, Huai'an, China; ^2^ Department of Endocrinology, Huai'an First People's Hospital, Nanjing Medical University, Huai'an, China; ^3^ Department of Orthopedics, Huai'an First People's Hospital, Nanjing Medical University, Huai'an, China; ^4^ Clinical Laboratory, Huai'an First People's Hospital, Nanjing Medical University, Huai'an, China

**Keywords:** dexamethasone, osteoblasts, miR-200a, keap1, Nrf2

## Abstract

Treatment with dexamethasone in human osteoblasts leads to oxidative stress and cell injures. NF-E2-related factor 2 (Nrf2) is a key anti-oxidant signaling. We want to induce Nrf2 activation via microRNA-mediated silencing its suppressor Keap1. Our results show that microRNA-200a (“miR-200a”) expression depleted Keap1, causing Nrf2 protein stabilization in OB-6 osteoblastic cells. Reversely, the miR-200a anti-sense led to Keap1 upregulation and Nrf2 degradation. miR-200a expression activated Nrf2 signaling, which inhibited dexamethasone-induced reactive oxygen species production and OB-6 cell death/apoptosis. Keap1 shRNA also activated Nrf2 and protected OB-6 cells from dexamethasone. Importantly, miR-200a was in-effective in Keap1-silenced (by shRNA) OB-6 cells. In the primary human osteoblasts, Keap1 silence by targeted-shRNA or miR-200a protected cells from dexamethasone. Significantly, miR-200a level was decreased in necrotic femoral head tissues, which was correlated with Keap1 mRNA upregulation. Together, miR-200a expression activates Nrf2 signaling and protects human osteoblasts from dexamethasone.

## INTRODUCTION

Existing literatures have demonstrated that dexamethasone (Dex) and other glucocorticoids shall exert direct injuries to human osteoblasts [1–3]. Osteoblast cell apoptosis is frequently detected in the bones of Dex-taking patients [4, 5]. Our group [6–12] and others [13–15] have been treating human osteoblasts/osteoblastic cells with Dex, which mimicked glucocorticoid-induced injuries [4, 7, 8, 16, 17]. This cellular model would help us to investigate the underlying mechanism of Dex-induced cell injuries [6–9, 11, 12].

We [7–9, 11] have shown that Dex induces reactive oxygen species (ROS) production, which induces apoptosis in human osteoblasts/osteoblastic cells [9, 16]. On the other hand, ROS clearance effectively protects osteoblasts/osteoblastic cells from Dex [16–18]. NF-E2-related factor 2 (Nrf2) signaling is arguably one of the most important cellular defense mechanism against oxidative stress [19, 20]. Keap1 (Kelch-like erythroid cell-derived protein with CNC homology [ECH]-associated protein 1) is the key regulator and repressor of the Nrf2 [19, 20]. In the resting condition, Keap1 associates with Nrf2 to dictate Nrf2 ubiquitination and degradation [19, 20]. On the other hand, Keap1 silence, mutation or in-activation could lead to Nrf2 stabilization [21, 22]. The latter translocates to nuclei, where it dictates transcription of multiple anti-oxidant genes via binding to antioxidant response element (ARE) [19, 20].

miRNAs (miRs) inhibit targeted-gene expression via binding to 3′-untranslated region (UTR) of the mRNAs [23–25]. Our results here demonstrate that microRNA-200a (“miR-200a”) targets Keap1 mRNA. More importantly, forced-expression of miR-200a silences Keap1 to activate Nrf2 signaling, which protects human osteoblasts/osteoblastic cells from Dex.

## RESULTS

### miR-200a expression depletes Keap1 to cause Nrf2 accumulation in human osteoblastic cells

**Figure 1:**
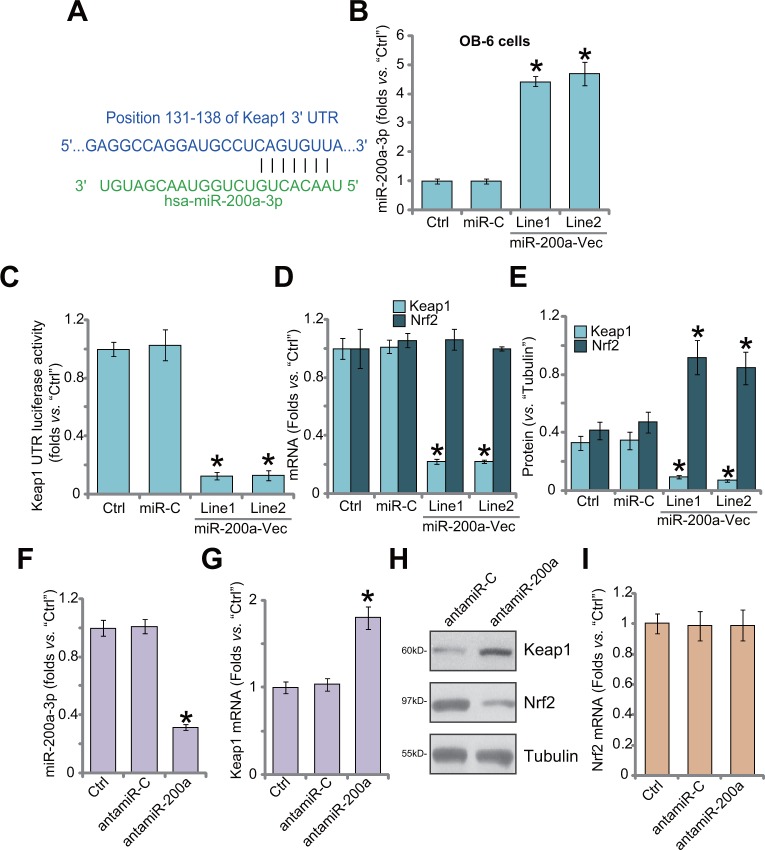
miR-200a expression depletes Keap1 to cause Nrf2 accumulation in human osteoblastic cells MicroRNA-200a-3p (“miR-200a-3p”) putatively targets the 3′-UTR of Keap1 mRNA **(A)**. Stable OB-6 osteoblastic cells, expressing miR-200a expression vector (“miR-200a-Vec”, two lines, “Line1/2”), non-sense microRNA-control (“miR-C”), or the parental control OB-6 cells (“Ctrl”) were subjected to quantitative real-time PCR (“qRT-PCR”) assay **(B and D)**, Keap1 mRNA 3′-UTR luciferase activity assay **(C)** and Western blotting assay (**E**, results were quantified). Relative expression of miR-200a-3p **(F)**, Keap1 mRNA **(G)**, Keap1/Nrf2 protein **(H)** and Nrf2 mRNA **(I)** in stable OB-6 cells with miR-200a anti-sense (“antamiR-200a”), antagomiR-control (“antamiR-C”), as well as in parental control cells (“Ctrl”) were shown. Data were expressed as mean ± SD (n=5). ^*^ *p* <0.05 *vs.* “Ctrl”. Experiments in this figure were repeated three times, and similar results were obtained.

First, we demonstrate that miR-200a (“-3p”) putatively targets the 3′-UTR of Keap1 mRNA (at position 131-138) (Figure 1A), as reported by other studies [26, 27]. Next, the miR-200a expression vector (pSuper-GFP-puro) was established, which was transfected to OB-6 human osteoblastic cells [28, 29]. Via puromycin selection, two stable OB-6 cell lines (“Line1/2”) with the construct were established. As shown in Figure 1B, miR-200a-3p level was significantly increased in stable cells. Remarkably, 3′-UTR luciferase activity of Keap1 mRNA was dramatically decreased in the miR-200a-expressing cells (Figure 1C). Consequently, Keap1 mRNA (Figure 1D) and protein (Figure 1E) expression was also reduced. Nrf2 mRNA expression was unchanged after miR-200a expression in OB-6 cells (Figure 1D), yet Nrf2 protein was stabilized and accumulated (Figure 1E). The non-sense microRNA-control (“miR-C”) failed to change expressions of miR-200a (Figure 1B) and Keap1/Nrf2 (Figure 1D–1E). Thus, miR-200a expression depletes Keap1 to cause Nrf2 stabilization.

Next, the miR-200a anti-sense (“antamiR-200a”) was introduced to OB-6 cells. AntamiR-200a indeed decreased miR-200a-3p expression (Figure 1F). As a result, Keap1 mRNA (Figure 1G) and protein (Figure 1H) expressions were upregulated. Consequently, Nrf2 protein was downregulated (Figure 1H). Expression of Nrf2 mRNA was unchanged (Figure 1I), confirming Nrf2 protein degradation. The antagomiR-control (“antamiR-C”) failed to change expressions of miR-200a, Keap1 nor Nrf2 (Figure 1F–1H). Therefore, miR-200a depletion by anti-sense induces Keap1 upregulation and Nrf2 degradation.

### miR-200a expression activates Nrf2 signaling and protects human osteoblastic cells from Dex

**Figure 2:**
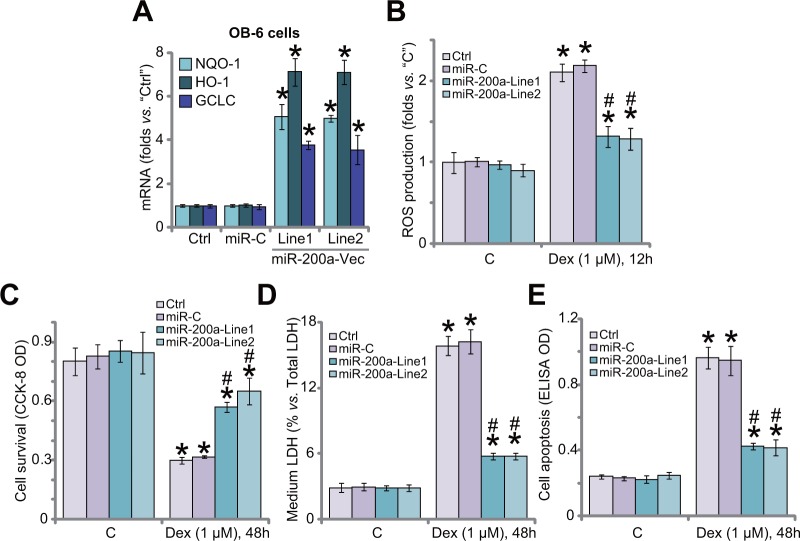
miR-200a expression activates Nrf2 signaling and protects human osteoblastic cells from Dex Stable OB-6 cells, expressing miR-200a expression vector (two lines, “Line1/2”), non-sense microRNA-control (“miR-C”), or the parental control cells (“Ctrl”) were treated with/out Dex (1 μM) for applied time; Relative expressions of listed genes were tested by quantitative real-time PCR (“qRT-PCR”) assay **(A)**; ROS level was presented in **(B)**; Cell viability (CCK-8 assay, **(C)**, cell death (LDH release assay, **(D and F)** and apoptosis (Histone DNA ELISA assay, **(E)** were also tested. Data were expressed as mean ± SD (n=5). “C” stands for no Dex treatment. ^*^ *p* <0.05 *vs.* “C”/“Ctrl”. ^#^ *p* <0.05 *vs.* Dex treatment in “miR-C” cells. Experiments in this figure were repeated three times, and similar results were obtained.

In the resting condition, Keap1 binds to Nrf2 to cause Nrf2 ubiquitination and degradation [30]. Above results have shown that miR-200a expression silenced Keap1 to cause Nrf2 stabilization and accumulation in OB-6 cells. We therefore tested Nrf2-dependent genes, including heme oxygenase-1 (HO1), NADPH quinone oxidoreductase 1 (NQO1) and glutamate cysteine ligase catalytic subunit (GCLC), in these cells. Quantitative real-time PCR (“qRT-PCR”) assay results in Figure 2A showed that mRNA expressions of the Nrf2 genes (HO1, NOQ1 and GCLC) were significantly increased in OB-6 cells expressing miR-200a, indicating Nrf2 signaling activation. We have previously shown that ROS production and oxidative stress are major contributors of Dex-induced osteoblast injuries [7–9, 11]. Nrf2 is the well-established and vital anti-oxidant signaling [19, 31–33]. Here, we showed that Dex-induced ROS production was largely attenuated after expression of miR-200a in OB-6 cells (Figure 2B). Remarkably, Dex-induced OB-6 cell viability reduction (CCK-8 OD/optic density, Figure 2C), cell death (LDH release, Figure 2D) and apoptosis (Histone DNA apoptosis ELISA OD, Figure 2E) were significantly alleviated in miR-200a-expressing cells. The non-sense microRNA-control (“miR-C”) failed to change Nrf2 signaling (Figure 2A), ROS production (Figure 2B) nor cell survival (Figure 2C–2E) in Dex-treated cells. Together, these results suggest that miR-200a expression activates Nrf2 cascade to inhibit oxidative stress, and protects OB-6 cells from Dex.

### Keap1 shRNA induces Nrf2 stabilization in human osteoblastic cells

**Figure 3:**
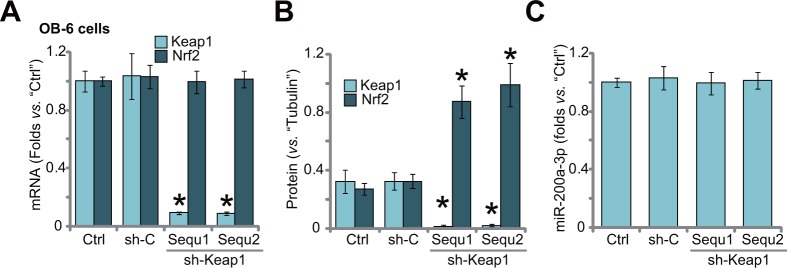
Keap1 shRNA induces Nrf2 stabilization in human osteoblastic cells Stable OB-6 cells expressing Keap1 shRNA (sh-Keap1, “Sequ1/2”), non-sense control shRNA (“sh-C”), or the parental control cells (“Ctrl”) were subjected of qRT-PCR assay **(A and C)** and Western blotting assay (Data were quantified in **(B)**, relative Keap1/Nrf2 expression and miR-200a-3p level were shown. Data were expressed as mean ± SD (n=5). ^*^ *p* <0.05 *vs.* “Ctrl”. Experiments in this figure were repeated three times, and similar results were obtained.

If Keap1 is the primary target of miR-200a in OB-6 cells, then direct silence of Keap1 should also protect cells from Dex. Thus, shRNA method was utilized to knockdown Keap1. A set of two distinct lentiviral Keap1 shRNAs (sh-Keap1, “Sequ1/2”), with non-overlapping sequence, were applied. qRT-PCR assay results in Figure 3A demonstrated that Keap1 mRNA was dramatically downregulated after stably expressing the shRNA. On the other hand, Nrf2 mRNA level was unchanged (Figure 3A). Keap1 protein was also significantly reduced in the OB-6 cells (Figure 3B). Notably, Keap1 knockdown induced Nrf2 stabilization/accumulation (Figure 3B). As expected, Keap1 shRNA didn't change the level of miR-200a-3p in OB-6 cells (Figure 3C). Also, non-sense shRNA control (“sh-C”) had on significant effect on expressions of Keap1/Nrf2 (Figure 3A and 3B) nor miR-200a-3p (Figure 3C).

### Keap1 shRNA activates Nrf2 and protects human osteoblastic cells from Dex

**Figure 4:**
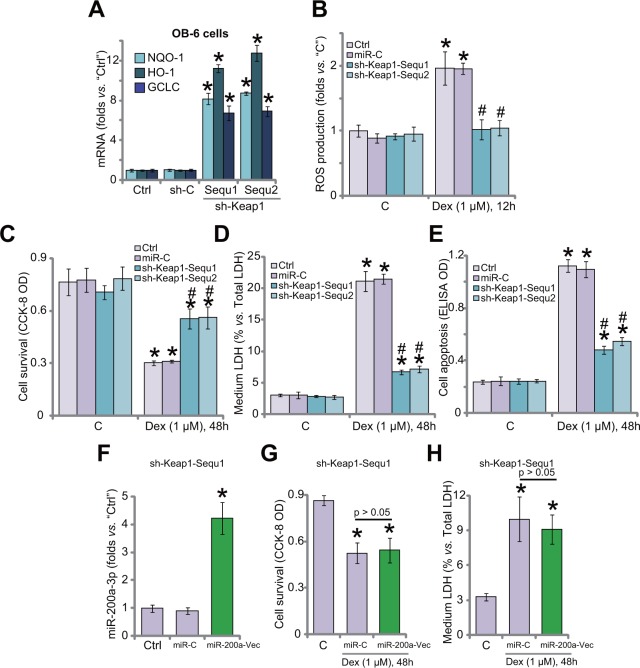
Keap1 shRNA activates Nrf2 and protects human osteoblastic cells from Dex Stable OB-6 cells expressing Keap1 shRNA (sh-Keap1, “Sequ1/2”), non-sense control shRNA (“sh-C”), or the parental control cells (“Ctrl”) were treated with/out Dex (1 μM) for applied time; Expressions of listed genes were tested by quantitative real-time PCR (“qRT-PCR”) assay **(A)**; Relative ROS level was shown in **(B)**; Cell viability (CCK-8 assay, **(C)**, cell death (LDH release assay, **(D)** and apoptosis (Histone DNA ELISA assay, **(E)** were examined. Stable OB-6 cells expressing Keap1 shRNA (sh-Keap1, “Sequ1”) were also transfected with/out miR-200a expression vector (“miR-200a-Vec”); Cells were then treated with/out Dex (1 μM) for applied time; MiR-200a-3p expression **(F)**, cell viability **(G)**, cell death **(H)** were tested. “C” stands for no Dex treatment. Data were expressed as mean ± SD (n=5). ^*^ *p* <0.05 *vs.* “C”/“Ctrl”. ^#^ *p* <0.05 *vs.* Dex treatment in “miR-C” cells. Experiments in this figure were repeated three times, and similar results were obtained.

Since Keap1 shRNA induced Nrf2 stabilization in OB-6 cells, expressions of Nrf2-regulaed genes were also tested. qRT-PCR assay results in Figure 4A confirmed that mRNA expressions of HO1, NQO1 and GCLC were significantly increased in Keap1-silenced OB-6 cells, where Dex-induced ROS production was largely inhibited (Figure 4B). Remarkably, Keap1-silenced OB-6 cells were protected from Dex (Figure 4C–4E). Dex-induced OB-6 cell viability reduction (Figure 4C), cell death (Figure 4D) and apoptosis (Figure 4E) were attenuated after Keap1 knockdown. Non-sense shRNA control (“sh-C”) didn't change Nrf2 signaling (Figure 4A), ROS production (Figure 4B) and cell survival (Figure 4C–4E) in Dex-treated OB-6 cells.

If Keap1 is the primary target of miR-200a, then miR-200a should be ineffective in Keap1-silenced cells. To test this hypothesis, miR-200a vector was introduced to Keap1 shRNA (“Sequ1”)-expressing OB-6 cells (Figure 4F). MiR-200a (−3p) level was again increased after expressing the construct in Keap1-silenced cells (Figure 4F). Remarkably, miR-200a expression was unable to further protect OB-6 cells from Dex, when Keap1 was already silenced by targeted-shRNA (“Sequ1”, Figure 4G and 4H). Thus, miR-200a expression was very much invalid against Dex when Keap1 was already silenced (Figure 4G and 4H). These results suggest that Keap1 should be the primary target of miR-200a in mediating its pro-survival function in OB-6 cells.

### Keap1 silence by shRNA or miR-200a protects primary human osteoblasts from Dex

**Figure 5:**
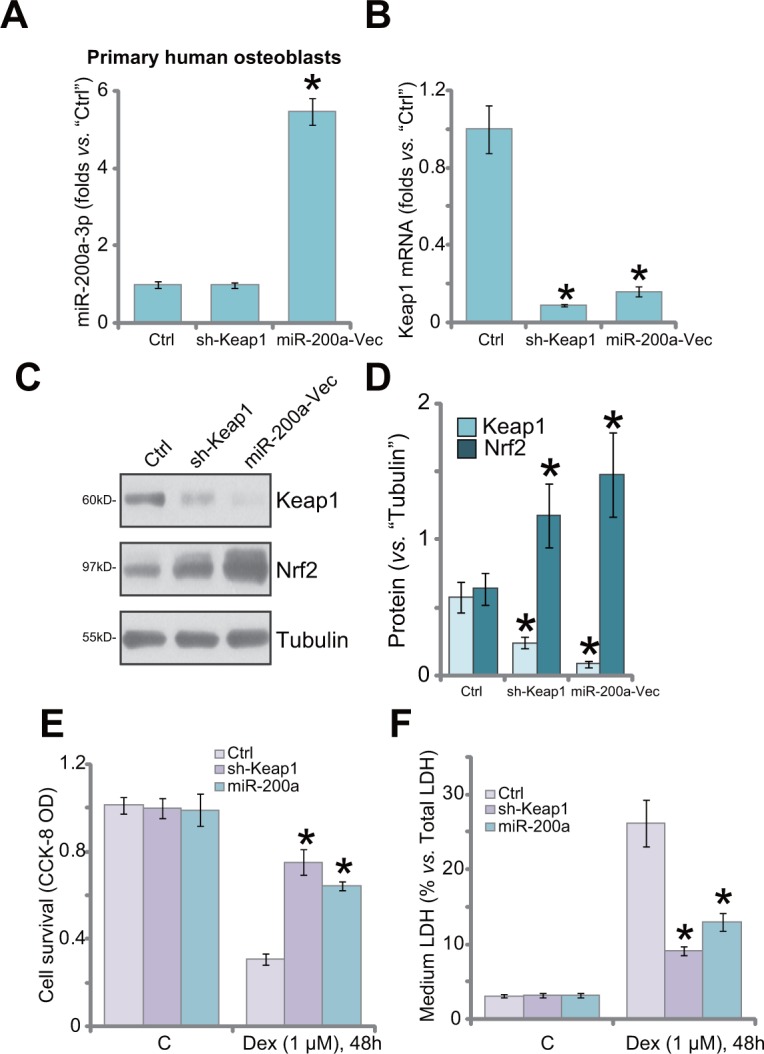
Keap1 silence by shRNA or miR-200a protects primary human osteoblasts from Dex Primary human osteoblasts were transfected with Keap1 shRNA (“Sequ1”) or miR-200a Vector (“miR-200a-Vec”) as described; Relative expression of miR-200a-3p **(A)**, Keap1 mRNA **(B)** and Keap1/Nrf2 protein **(C)**, and quantified in **(D)** were shown; Cells were also treated with/out Dex (1 μM) for 48 hours, cell viability (CCK-8 OD, **(E)** and cell death (LDH release, **(F)** were tested. “C” stands for no Dex treatment. “Ctrl” stands for un-transfected control cells. Data were expressed as mean ± SD (n=5). ^*^ *p* <0.05 *vs.* “Ctrl”.

Next, we focused the potential effect of miR-200a in the primary human osteoblasts. The primary cells were also transfected with miR-200a expression vector or Keap1-shRNA (“Sequ1”). qRT-PCR assay results showed that miR-200a (−3p) level was increased significantly only after introduction of the miR-200a construct (Figure 5A). Keap1 mRNA level was sharply decreased after expressing miR-200a or Keap1-shRNA (Figure 5B). Consequently, Keap1 protein downregulation and Nrf2 stabilization were observed in these cells (Figure 5C and 5D). Importantly, the primary human osteoblasts were obviously protected from Dex when Keap1 was silenced (Figure 5E and 5F). Dex-induced viability reduction (Figure 5E) and cell death (Figure 5F) were largely attenuated in osteoblasts with Keap1 shRNA or miR-200a.

### miR-200a downregulation and Keap1 mRNA upregulation in human necrotic femoral head tissues

**Figure 6:**
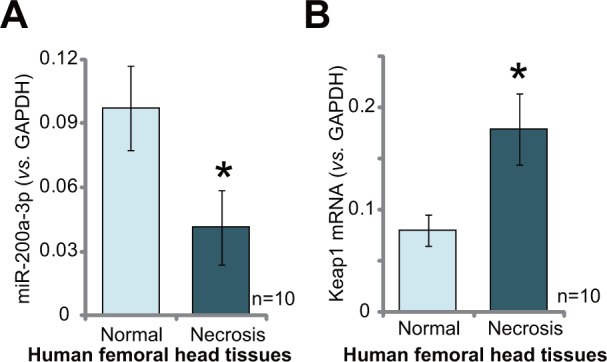
miR-200a downregulation and Keap1 mRNA upregulation in human necrotic femoral head tissues Relativeexpressions of miR-200a-3p **(A)** and Keap1 mRNA **(B)** in necrotic femoral head tissues (“Necrosis”) and surrounding normal femoral head tissues (“Normal”) from ten Dex-taking patients were shown. Data were expressed as mean ± SD (n=10). ^*^ *p* <0.05 *vs.* “Normal” tissues.

At last, we tested expression of miR-200a in human necrotic femoral head tissues. A total of 10 different necrotic femoral head tissues (“Necrosis”) from Dex-taking patients were tested. As compared to the surrounding normal femoral head tissues (“Normal”), expression of miR-200a (−3p) was significantly decreased in the “Necrosis” tissues (Figure 6A). Reversely, Keap1 mRNA was increased in the “Necrosis” tissues (Figure 6B). The miR-200a downregulation in human necrotic femoral head tissues indicates a potential function of this miRNA in the pathogenesis of femoral head necrosis in the Dex-taking patients.

## DISCUSSION

Recent studies have suggested that Dex-induced ROS production and subsequent oxidative stress are the major reason of osteoblast injuries [7, 9, 11, 13, 34]. On the other hand, inhibition of ROS could efficiently protect osteoblasts from Dex [7, 9, 11, 13, 34]. For instance, compound 13 (“C13”), a novel AMP-activated protein kinase (AMPK) activator [35], attenuated ROS production, and protected osteoblastic cells from Dex [9]. C13 increased (nicotinamide adenine dinucleotide phosphate) NADPH content to significantly attenuate oxidative stress in Dex-treated osteoblasts [9]. Further, Icariside II activated EGFR-Akt-Nrf2 anti-oxidant pathway to protect osteoblasts from Dex [11]. SC79, an Akt activator, protected osteoblasts from Dex also via decreasing ROS [34]. Therefore, ROS clearance is a fine strategy to protect osteoblasts from Dex.

The transcription factor Nrf2 dictates a genetic program which protects cells from oxidative stress and maintains cellular redox homeostasis [19, 20, 36, 37]. Keap1 is a BTB-Kelch protein and the major upstream suppressor of Nrf2 [19, 20, 36, 37]. Keap1 is vital for Nrf2′s subcellular localization and steady-state level [19, 20, 36, 37]. Keap1 functions as an E3 ubiquitin ligase complex with Cul3 and Rbx1, which triggers Nrf2 ubiquitin conjugation and degradation [19, 20, 36, 37]. Keap1 silence, mutation or in-activation shall cause Nrf2 stabilization and accumulation, which then activates Nrf2 cascade [19, 20, 36, 37].

Very recent studies have identified potential miRNAs that could possibly activate Nrf2 via depleting Keap1. For example, Yang *et al.*, showed that miR-200a activates Nrf2 by targeting Keap1 in hepatic stellate cells [38]. Shi *et al.*, showed that miR-141 activates Nrf2-dependent antioxidant pathway also via depleting Keap1 [39]. Similarly, a very recent study by Gong *et al.*, showed that miRNA-141 attenuated UV-induced oxidative stress by activating Keap1-Nrf2 signaling [40]. Therefore, miR-mediated silence of Keap1 is effective in activating Nrf2 signaling.

In the current study, we demonstrate that miR-200a activated Nrf2 signaling in OB-6 cells and primary human osteoblasts. First, forced-expression of miR-200a induced Keap1 degradation, causing Nrf2 stabilization in human osteoblasts/osteoblastic cells. Reversely, miR-200a depletion by the antamiR-200a led to Keap1 upregulation and Nrf2 degradation. Second, stabilized Nrf2 induced transcription of several Nrf2-regulated genes (HO1, NOQ1 and GCLC) in miR-200a-expressing cells. Significantly, expression of miR-200a, by inducing Nrf2 activation, largely inhibited Dex-induced oxidative stress and subsequent osteoblasts/osteoblastic cell death and apoptosis. Third, Keap1 shRNA, mimicking miR-200a, activated Nrf2 and protected OB-6 cells from Dex. More importantly, miR-200a was unable to further protect OB-6 cells from Dex when Keap1 was silenced by shRNA. We therefore conclude that Keap1 is the primary target of miR-200a in osteoblasts in mediating its anti-Dex functions. Significantly, we found that miR-200a level was decreased in necrotic femoral head tissues, which was correlated with Keap1 mRNA upregulation. It will be interesting to further test the potential function of miR-200a on Dex-damaged human osteoblasts *in vivo*.

## MATERIALS AND METHODS

### Chemicals and reagents

Dex and puromycin were purchased from Sigma Aldrich (Nantong, China). Cell culture reagents were from Gibco (Nantong, China). Antibodies of this study were purchased from Cell Signaling Technology (Nanjing, China).

### Culture of human osteoblastic cells

The OB-6 human osteoblastic cells [4], obtained from the Cell Bank of Shanghai Institute of Biological Science (Shanghai, China), were described as described previously [10].

### Culture of primary human osteoblasts

The primary human osteoblasts were achieved from the redundant trabecular bone fragments of healthy donors. The trabecular bone fragments were minced into small pieces and washed. The bone pieces were then digested with 2 mg/mL collagenase type II (300 U/mg; Sigma) for 2 hours, which were placed in culture flasks with the described medium [41]. Medium was changed twice a week until cells reached confluence. Written-informed consent was obtained from each participant. The protocols were approved by Ethics Review Board of Nanjing Medical University.

### Human femoral head tissues

A total of 10 Dex-taking patients undergoing femoral head surgery were included. The necrotic femoral head tissues along with paired surrounding normal femoral head tissues were obtained. Tissues were subjected to tissues lysis buffer (Biyuntian, Wuxi, China) incubation. Samples were stored in liquid nitrogen for further analysis. Written-informed consent was obtained from each participant. The protocols were approved by Ethics Review Board of Nanjing Medical University.

### Expression of miR-200a/antamiR-200a

The pSuper-GPF-puro-miR-200a expression vector, encoding miR-200a, was designed by Genepharm (Shanghai, China). The miR-200a anti-sense (“antamiR-200a”) was provide by Ambion (Shanghai, China). Human osteoblasts/osteoblastic cells were transfected with miR-200a expression vector, antamiR-200a, scramble miRNA control (“miR-C”) or scramble antagomiR control (antagomiR-C) via the Lipofectamine 2000 reagent (Invitrogen, Suzhou, China). Stable cells were selected by puromycin (0.5 μg/mL, Sigma) for 72 hours.

### Keap1 3′-UTR luciferase assay

As previously described [27], Keap1 mRNA 3′-UTR was amplified via the primers (5′-ACGTACGCTAGCGAAGCAGATTGACCAGCAGA-3′ and 5′-ACGTACCTCGAGATGCGATGGGCAAAGATTAC-3′), which was sub-cloned into pSuper-GPF-puro vector. The reporter plasmid was then utilized as template to generate a miR-200a response element. OB-6 cells were transfected with luciferase reporter plasmid with Renilla luciferase phGR-TK (Promega, Wuxi, China) using Lipofectamine 2000 reagent. The luciferase activity was measured 48 hours after transfection using the Dual-Luciferase reporter assay system (Promega).

### Cell survival assay

As described in our previous studies [7–11], the viability of human osteoblasts/osteoblastic cells was analyzed by the Cell Counting Kit-8 (CCK-8, Dojindo Laboratories, Kumamoto, Japan) assay kit.

### Cell apoptosis assay

The histone-DNA ELISA cell apoptosis plus kit (Roche, Nanjing, China) was applied to quantify cell apoptosis in human osteoblasts/osteoblastic cells [7–9].

### Cell death assay

As described [7–9], cell death was reflected by the medium release of lactate dehydrogenase (LDH), using a commercial available two-step LDH assay kit (Takara, Tokyo, Japan) [7].

### Western blot assay

The detailed protocol of Western blotting assay was descibred previously, and blot results were quantified via ImageJ software [7–9].

### Keap1 shRNA

The two lentiviral Keap1 shRNAs (“Sequ1/2”, with non-overlapping sequences) were designed and synthesized by Genepharm Co. (Shanghai, China). The shRNA was directly to cultured osteoblasts/osteoblastic cells (10 μL virus/1 mL medium, per well) for 24 hours. Puromycin (0.5 μg/mL, Sigma) was added afterwards to select stable cells for additional 72 hours. Keap1 knockdown was verified via qRT-PCR assay and Western blotting assay. Control cells were infected with lentiviral scramble control shRNA [10].

### RNA isolation and RT-PCR

RNA from human osteoblasts/osteoblastic cells or fresh femoral head tissues was extracted via RNeasy Midi Kit (Qiagen, Wuxi, China). For each sample, 500 ng of total RNA was reverse-transcribed via the RT-PCR kit (TOYOBO, Japan). Quantitative real-time PCR (“qRT-PCR”) was performed using the SYBR green kit [42, 43] through the ABI-7600 system (Applied Biosystems, Shanghai, China). The mRNA primers of Nrf2 genes, including *Nrf2, HO-1*, GCLC and *NQO1*, as well as *Keap1* and GAPDH were previously described [44–47]. In addition, microRNA was converted to cDNA from 500 ng of total RNA (per treatment) via the First-Strand Synthesis Kit (SABiosciences, Frederick, MD). Follow-up miR analysis was performed through qRT-PCR assay using the miR-200a primers (SABiosciences) [27]. miR-200a level was also normalized to GAPDH.

### ROS assay

As previously described [7–9, 11, 12], the dichloro-dihydro-fluorescein diacetate (DCFH-DA) fluorescent dye (Invitrogen, Shanghai, China) assay was performed to test cellular ROS content.

### Statistical analysis

All values were expressed as means ± standard deviation (SD). The statistical significance of differences among groups were determined by one-way analysis of variance (ANOVA) followed by the Tukey's post hoc multiple comparison tests. *p* < 0.05 was considered significant.

## CONCLUSIONS

MiR-200a expression activates Nrf2 signaling and protects human osteoblasts/osteoblastic cells from Dex. Keap1 is the primary target of miR-200a in mediating its actions.
